# Crystal structure and Hirshfeld surface analyses, inter­action energy calculations and energy frameworks of 2-(anthracen-10-yl)-1*H*-benzo[*d*]imidazole

**DOI:** 10.1107/S2056989025011594

**Published:** 2026-01-20

**Authors:** Naser E. Eltayeb, Yaseen A. Almehmadi, Tuncer Hökelek, Jamal Lasri, Aidan P. McKay

**Affiliations:** a Department of Chemistry Rabigh College of Science and Arts King Abdulaziz University,Jeddah 21589 Saudi Arabia; b Department of Chemistry, Faculty of Pure and Applied Sciences, International University of Africa, Khartoum 2469, Sudan; c King Fahd Medical Research Center King Abdulaziz University,Jeddah 21589 Saudi Arabia; d Department of Physics, Hacettepe University, 06800 Beytepe, Ankara, Türkiye; e EaStCHEM School of Chemistry, University of St Andrews, Fife KY16 9ST, United Kingdom; Katholieke Universiteit Leuven, Belgium

**Keywords:** 2-(anthracen-10-yl)-1*H*-benzo[*d*]imidazole, crystal structure, hydrogen bond, π-stacking, Hirshfeld surface

## Abstract

The benzimidazole and anthracene moieties in the title compound, C_21_H_14_N_2_, are oriented at a dihedral angle of 46.00 (2)°. In the crystal, N—H⋯N hydrogen bonds link the mol­ecules into infinite chains along the *b*-axis direction. In addition, C—H⋯π inter­actions contribute to the consolidation of the packing. A Hirshfeld surface analysis of the crystal structure indicates that the most important contributions to the crystal packing are from H⋯H (47.2%) and H⋯/C⋯H (39.4%) inter­actions. An energy-framework calculation indicates that the electrostatic and dispersion energies are the most important contributors to the packing.

## Abstract 2

The benzimidazole and anthracene moieties in the title compound contains are oriented at a dihedral angle of 46.00 (2)°. In the crystal, N—H⋯N hydrogen bonds link the mol­ecules into infinite chains along the *b*-axis direction. In addition, C—H⋯π inter­actions contribute to the consolidation of the packing.

## 1. Chemical context

Benzimidazole, a nitro­gen-containing aromatic heterocycle, is a key component in different biologically active mol­ecules (Bansal & Silakari, 2012 ▸) encompassing a broad spectrum of activities, including anti­cancer (Chen *et al.*, 2010 ▸; Sontakke *et al.*, 2015 ▸), anti­viral (Li *et al.*, 2006 ▸), anti­microbial (Sharma *et al.*, 2009 ▸) and anti­fungal (Goker *et al.*, 2002 ▸) activities. Different chemotherapeutic anti­cancer drugs inter­act directly with DNA or prevent the appropriate relaxation of DNA through inhibition of topoisomerases (Chen & Liu, 1994 ▸). Currently, our research work focuses on the synthesis, characterization and anti­cancer evaluation of a variety of acyclic and cyclic imine-type compounds (Eltayeb *et al.*, 2020*a* ▸,*b* ▸, 2025 ▸; Lasri *et al.*, 2018 ▸, 2023*a* ▸,*b* ▸, 2024 ▸, 2025 ▸). Herein we report the synthesis, mol­ecular and crystal structure, Hirshfeld surface analysis, inter­action energy calculations and energy frameworks of the title compound (I).

## 2. Structural commentary

**Figure 1:**
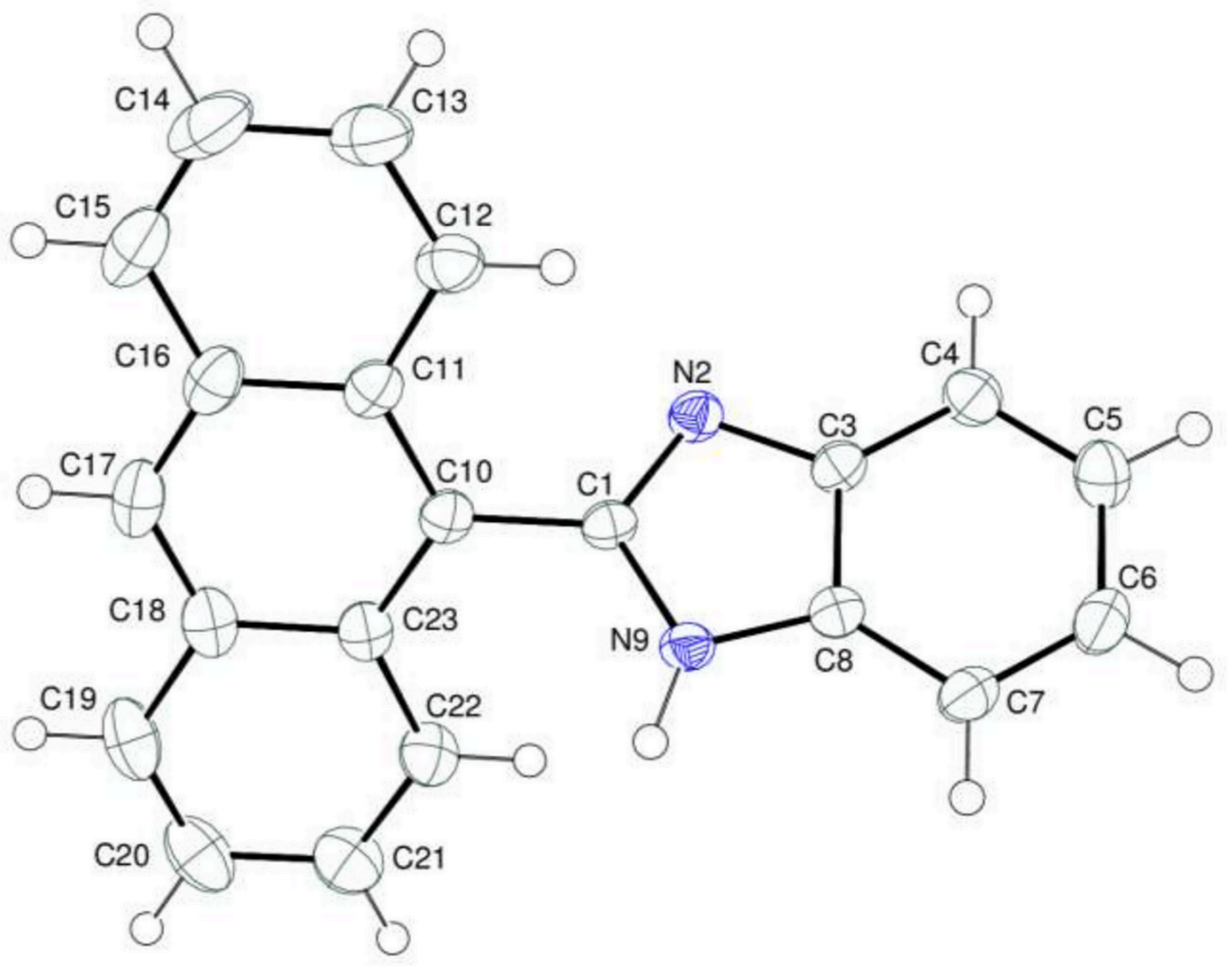
Mol­ecular structure of the title mol­ecule with atom-numbering scheme and 50% probability ellipsoids.

The title compound contains benzimidazole and anthracene ring systems (Fig. 1 ▸). The benzimidazole moiety is essentially planar [r.m.s. deviation = 0.013 (1) Å] with a maximum deviation of 0.0195 (12) Å for atom C5. In the anthracene moiety, the almost planar *A* (C11–C16), *B* (C10/C11/C16–C18/C23) and *C* (C18–C23) rings are oriented at dihedral angles of *A*/*B* = 2.48 (4)°, *A*/*C* = 5.67 (4)° and *B*/*C* = 3.20 (4)° and are thus nearly coplanar. The dihedral angle between the mean planes of the benzimidazole and anthracene ring systems is 46.00 (2)°. There are no unusual bond lengths or inter­bond angles in the mol­ecule.
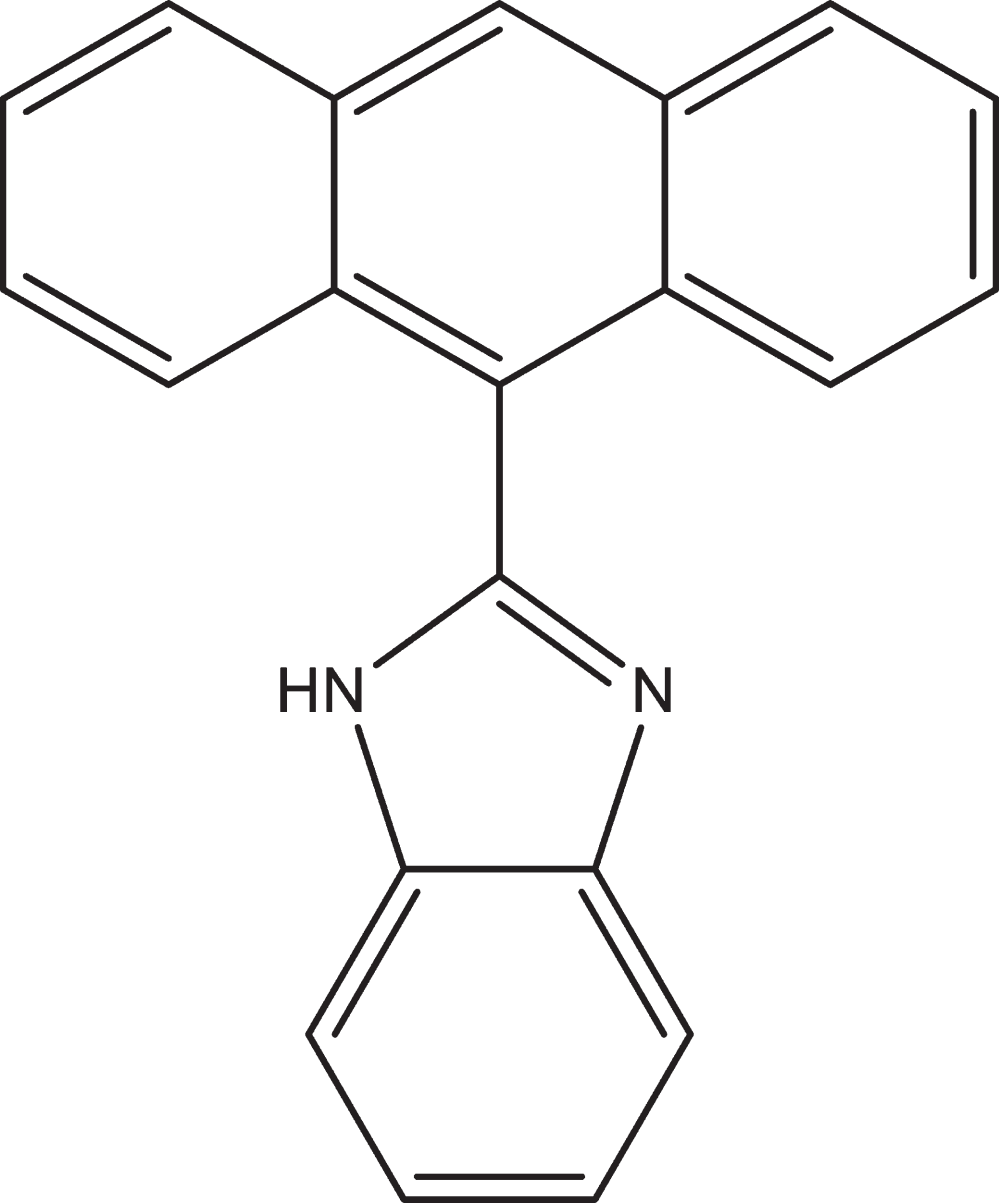
.

## 3. Supra­molecular features

**Figure 2:**
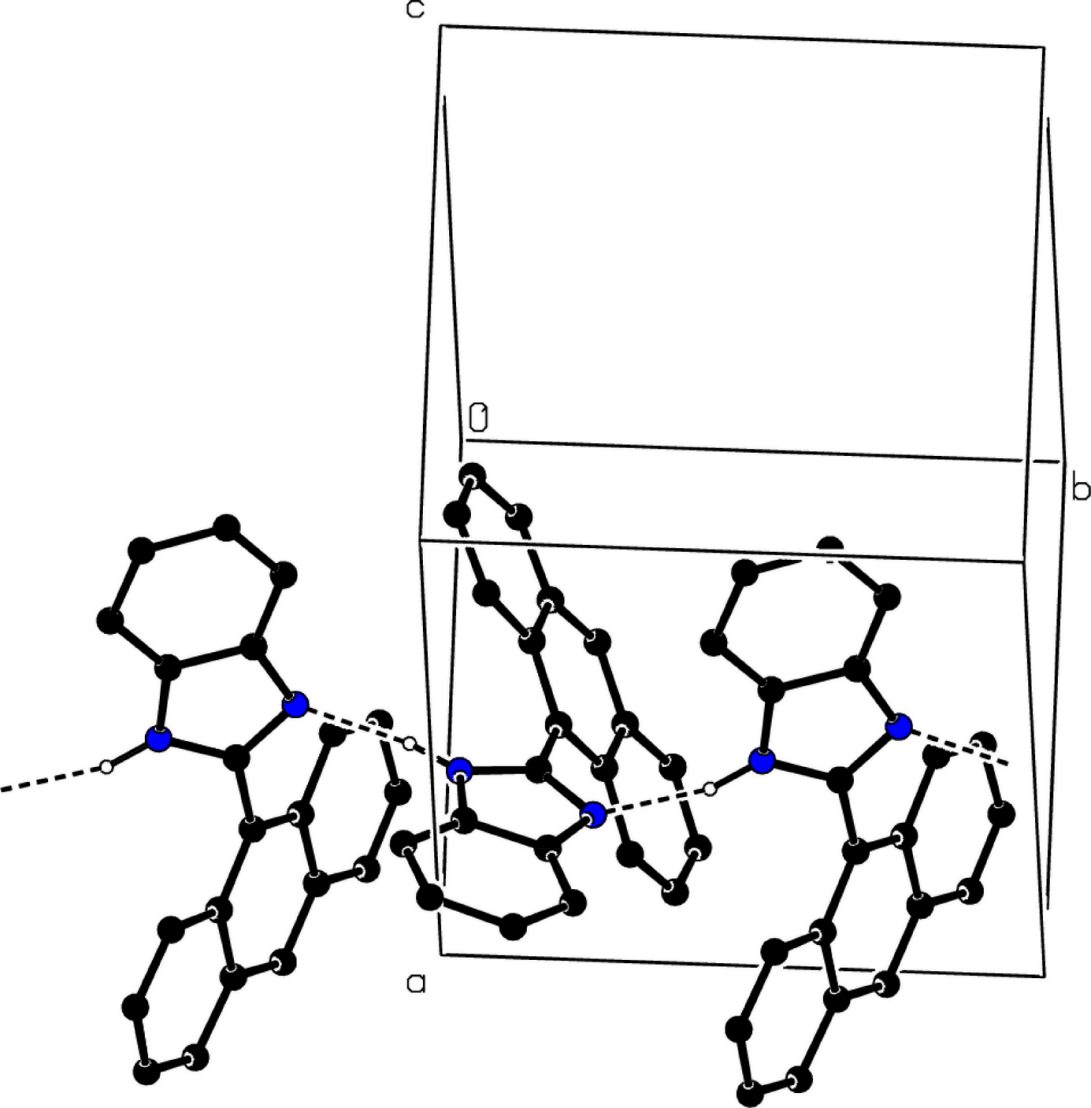
A partial packing diagram of the title compound. Inter­molecular N—H⋯N hydrogen bonds are shown as dashed lines. H atoms not involved in these inter­actions have been omitted for clarity.

In the crystal, N—H⋯N hydrogen bonds (Table 1 ▸) link the mol­ecules into infinite chains along the *b*-axis direction (Fig. 2 ▸). The C—H⋯π inter­actions (Table 1 ▸) help to consolidate the crystal packing. Despite the presence of aromatic rings, there are no π–π stacking inter­actions. The shortest centroid-to-centroid distances are 4.1943 (7) Å [for the *A* and *B* rings, α = 32.09 (3)°], 4.3273 (7) Å [for the (C3–C8) and *C* rings, α = 43.98 (4)°] and 4.4415 (7) Å [for the (N2/N9/C1/C3/C8) and *C* rings, α = 44.82 (4)°].

**Figure 3:**
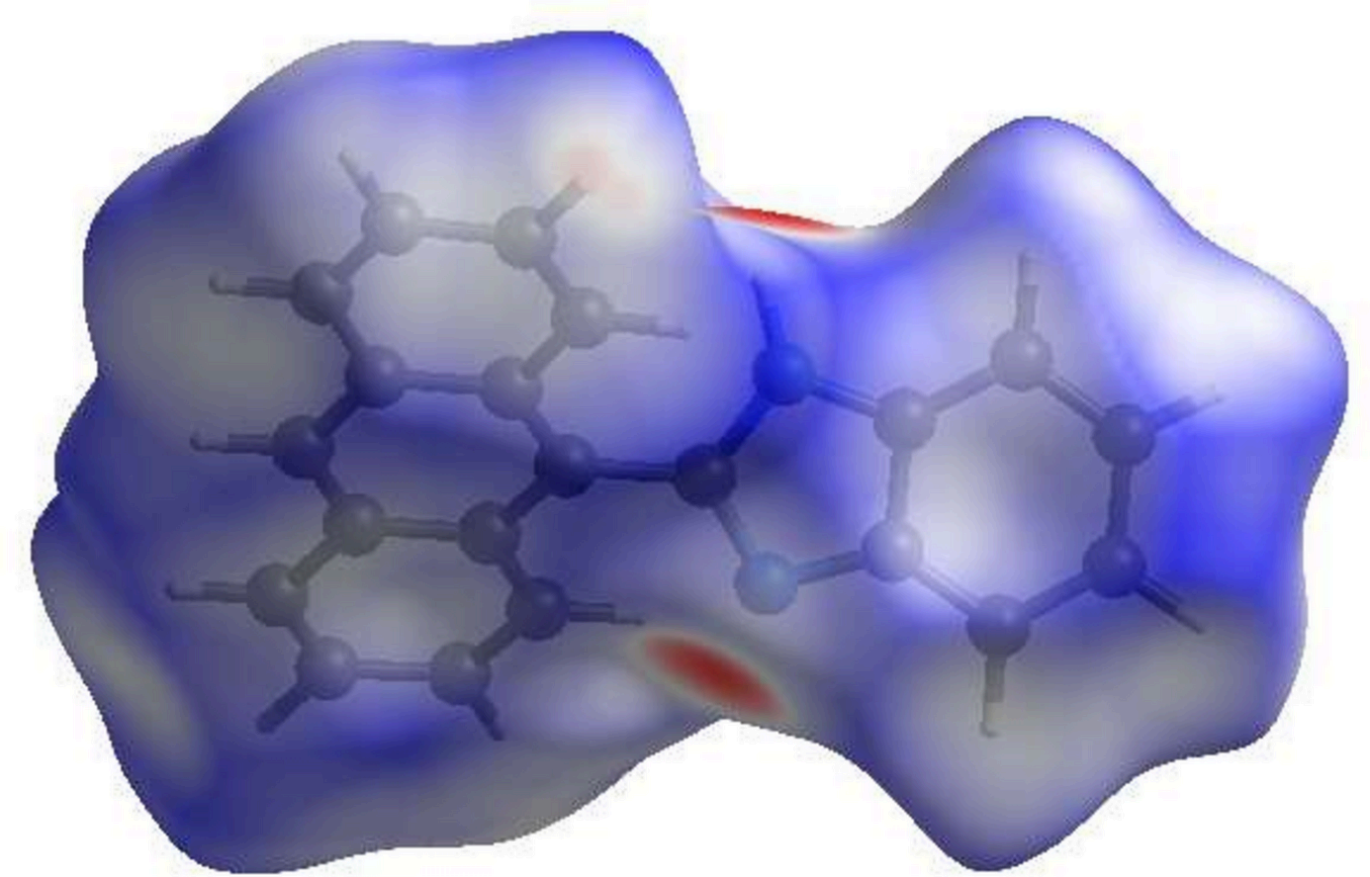
View of the three-dimensional Hirshfeld surface of the title compound plotted over *d*_norm._

**Figure 4:**
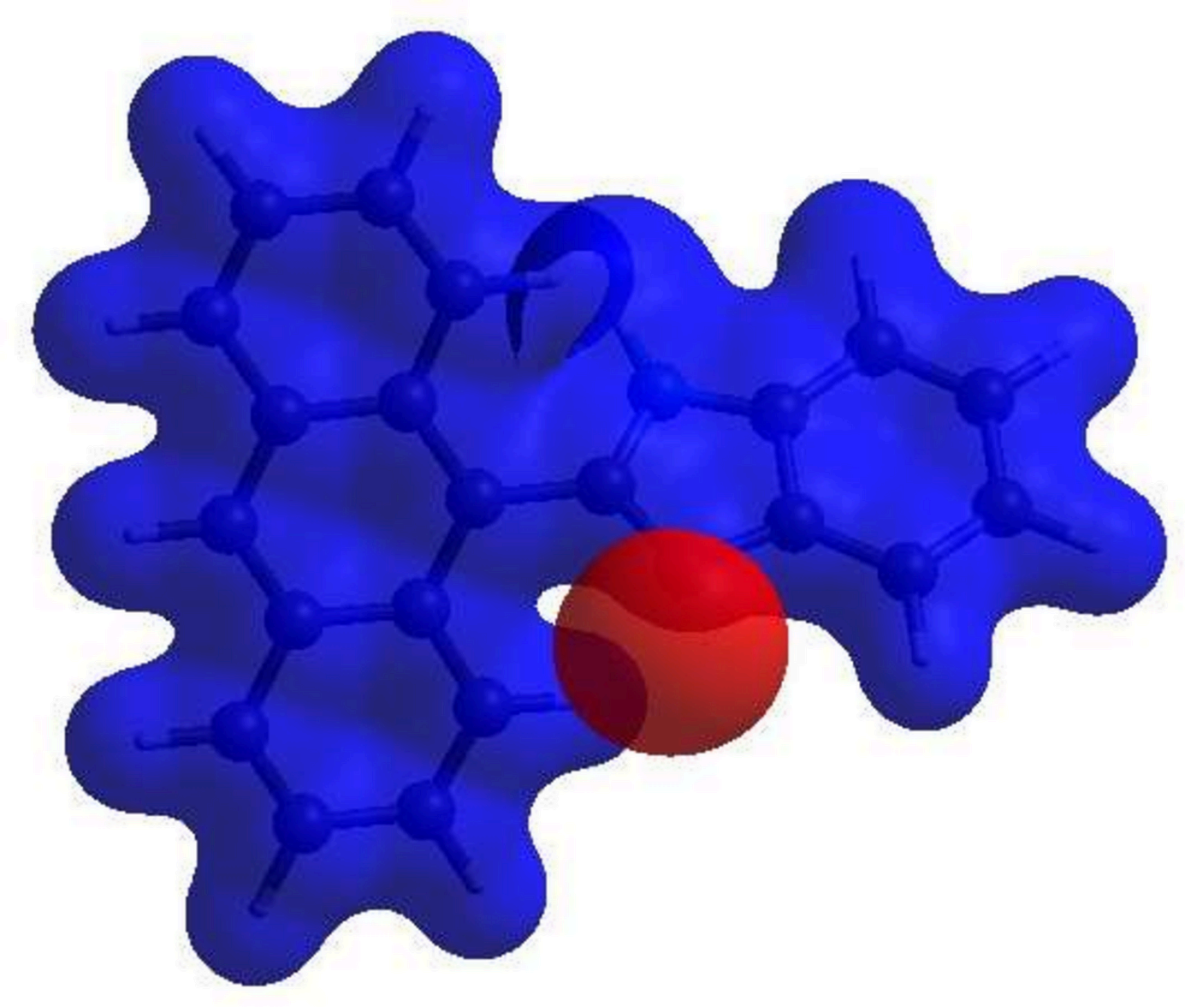
View of the Hirshfeld surface of the title compound plotted over electrostatic potential energy using the STO-3 G basis set at the Hartree–Fock level of theory. Hydrogen-bond donors and acceptors are shown as blue and red regions around the atoms corresponding to positive and negative potentials, respectively.

**Figure 5:**
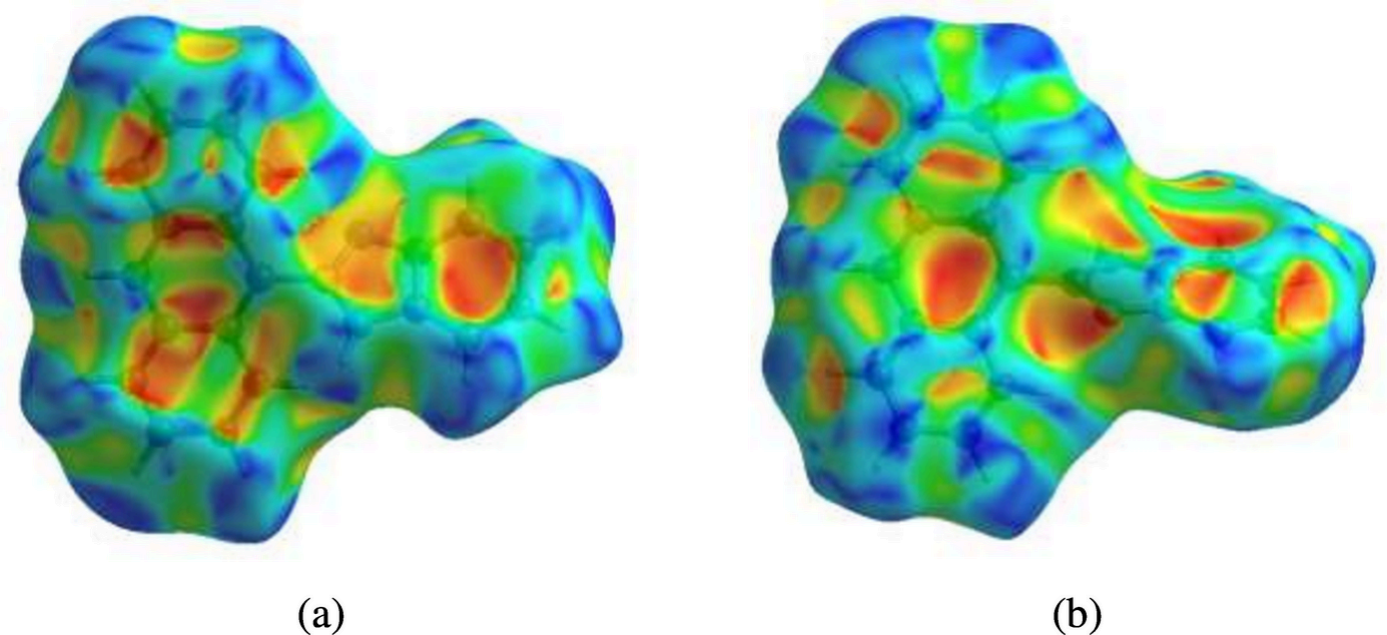
Hirshfeld surface of the title compound for two orientations plotted over shape-index.

**Figure 6:**
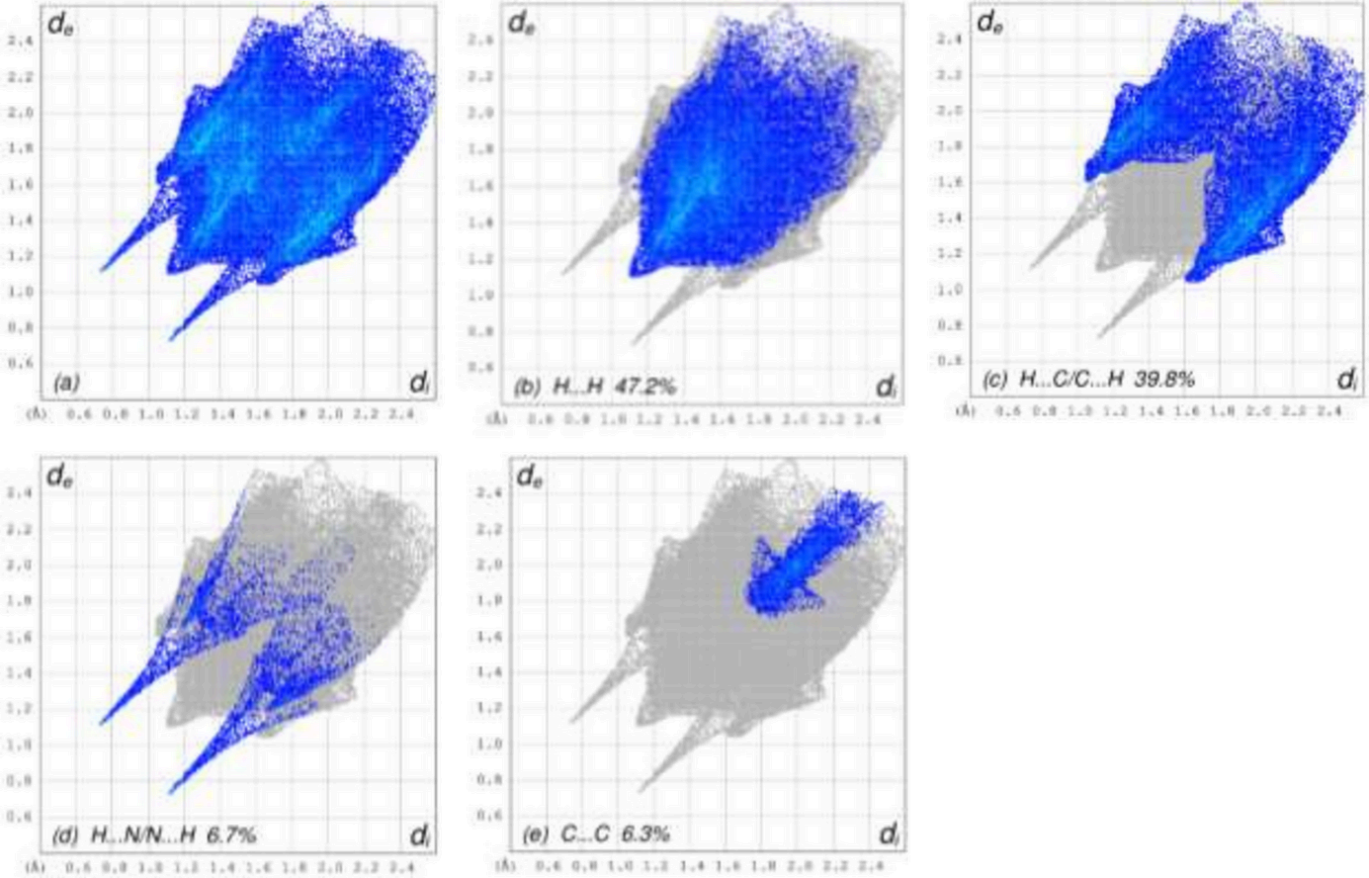
The full two-dimensional fingerprint plots for the title compound, showing (*a*) all inter­actions, and delineated into (*b*) H⋯H, (*c*) H⋯C/C⋯H, (*d*) H⋯N/N⋯H and (*e*) C⋯C inter­actions. The *d*_i_ and *d*_e_ values are the closest inter­nal and external distances (in Å) from given points on the Hirshfeld surface.

A Hirshfeld surface (HS) analysis was carried out using *Crystal Explorer 17.5* (Spackman *et al.*, 2021 ▸) to clarify the inter­molecular inter­actions in (I). The Hirshfeld surface plotted over *d*_norm_ is shown in Fig. 3 ▸, where the bright-red spots correspond to the respective donors and/or acceptors; they also appear as blue and red regions in Fig. 4 ▸ corresponding to positive and negative potentials (Spackman *et al.*, 2008 ▸). The absence of π–π stacking inter­actions is indicated by the absence of the adjacent red and blue triangles in the rings (Fig. 5 ▸*a* and *b*). On the other hand, the C—H⋯π inter­actions (Table 1 ▸) are represented as red π-holes, which are related to the electron ring inter­actions between C—H groups with the centroid of the *B* (C3–C8) and *C* (C10/C11/C16–C18/C23) rings of neighbouring mol­ecules (Fig. 5 ▸*a* and *b*). According to the two-dimensional fingerprint plots (McKinnon *et al.*, 2007 ▸), the inter­molecular H⋯H and H⋯C/C⋯H (Table 2 ▸) contacts make the most important contributions to the HS (47.2% and 39.8%, respectively) (Fig. 6 ▸).

## 4. Inter­action energy calculations and energy frameworks

**Figure 7:**
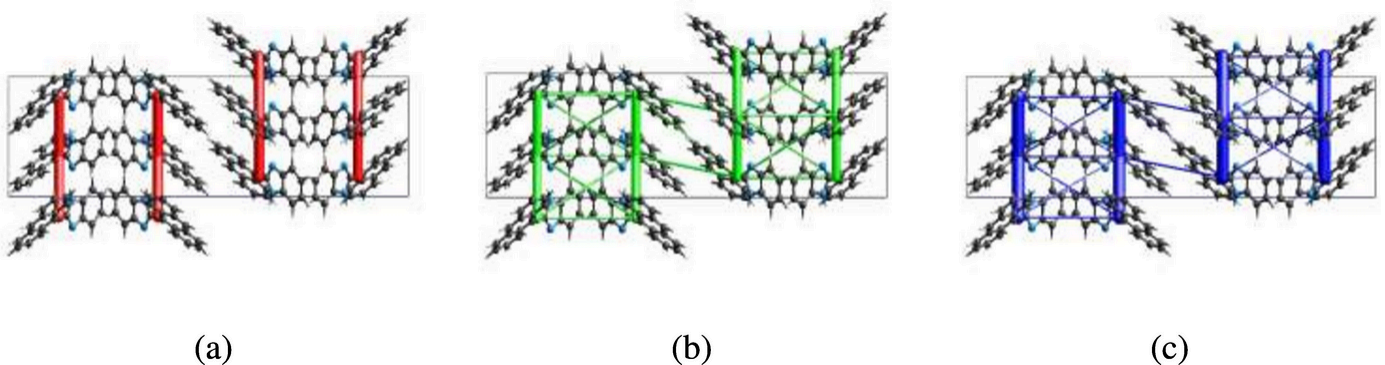
The energy frameworks for a cluster of mol­ecules of the title compound viewed down the *a*-axis showing the (*a*) electrostatic energy, (*b*) dispersion energy and (*c*) total energy diagrams. The cylindrical radius is proportional to the relative strength of the corresponding energies and they were adjusted to the same scale factor of 80 with cut-off value of 5 kJ mol^−1^ within 2 × 2 × 2 unit cells.

The CE–B3LYP/6–31G(d,p) energy model available in *Crystal Explorer 17.5* (Spackman *et al.*, 2021 ▸) was used to calculate the inter­molecular inter­action energies. Hydrogen-bonding inter­action energies (in kJ mol^−1^) for N9—H9⋯N2 were calculated to be −57.9 (*E*_ele_), −16.4 (*E*_pol_), −60.9 (*E*_dis_), 86.9 (*E*_rep_) and −72.7 (*E*_tot_). Energy frameworks combine the calculation of inter­molecular inter­action energies with a graphical representation of their magnitude (Turner *et al.*, 2015 ▸). Energy frameworks were constructed for *E*_ele_ (red cylinders), *E*_dis_ (green cylinders) and *E*_tot_ (blue cylinders) (Fig. 7 ▸*a*, *b* and *c*), and their evaluation indicates that the stabilization is dominated equally *via* the electrostatic and dispersion energy contributions in the crystal structure of (I).

## 5. Synthesis and crystallization

To a solution of 9-anthracenecarboxaldehyde (206.2 mg, 1.0 mmol) in ethanol (50 ml) was added 1,2-phenyl­enedi­amine (108.1 mg, 1.0 mmol) and the reaction mixture was refluxed for 4 h. The reaction was cooled to room temperature for precipitation, and then filtered. Yellow crystals suitable for X-ray analysis were obtained by slow evaporation of an ethanol solution. Yield: 70%. M.p. 534–536 K. IR (cm^−1^): 1227, 1401, 1619, 2917, 3050. ^1^H NMR (DMSO-*d*_6_): δ 7.31 (*s*, 2H), 7.50 (*m*, 4H), 7.71 (*d*, *J* = 8.1 Hz, 4H), 8.18 (*d*, *J* = 7.2 Hz, 2H), 8.79 (*s*, 1H), 13.02 (*bs*, 1H). ^13^C NMR (DMSO-*d*_6_): δ 121.9, 125.4, 125.6, 126.6, 128.3, 128.7, 130.4, 130.5, 149.4. Elemental analysis calculated for C_21_H_14_N_2_ (294.36), C, 85.69; H, 4.79; N, 9.52%. Found: C, 85.68, H, 4.77, N, 9.51%. This compound has been previously synthesized by Sontakke *et al.* (2015 ▸) and Barwiolek *et al.* (2019 ▸).

## 6. Refinement

Crystal data, data collection and structure refinement details are summarized in Table 2 ▸. The N-bound H atom was located in a difference Fourier map and was refined isotropically. The C-bound H atoms were calculated geometrically at a distance of 0.95 Å (for aromatic CH) and refined using a riding model by applying the constraint *U*_iso_(H) = 1.2*U*_eq_(C).

## Supplementary Material

Crystal structure: contains datablock(s) I. DOI: 10.1107/S2056989025011594/vm2321sup1.cif

Structure factors: contains datablock(s) I. DOI: 10.1107/S2056989025011594/vm2321Isup2.hkl

CCDC reference: 2518388

Additional supporting information:  crystallographic information; 3D view; checkCIF report

## Figures and Tables

**Table 1:** Hydrogen-bond geometry (Å, °) *Cg*1, *Cg*2, *Cg*3 and *Cg*4 are the centroids of the (N2/N9/C1/C3–C8), (C10/C11/C16–C21), (C10/C11/C16–C18,C23) and (C3–C8) rings, respectively.

*D*—H⋯*A*	*D*—H	H⋯*A*	*D*⋯*A*	*D*—H⋯*A*
N9—H9⋯N2^i^	0.95 (1)	1.92 (1)	2.8186 (13)	157 (1)
C5—H5⋯*Cg*1^iii^	0.95	2.96	3.8219 (12)	151
C13—H13⋯*Cg*2^iv^	0.95	2.97	3.4156 (14)	110
C20—H20⋯*Cg*3^i^	0.95	2.90	3.5625 (16)	128
C21—H21⋯*Cg*4^v^	0.95	2.75	3.2887 (15)	116

Symmetry codes: (i) 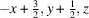; (iii) 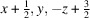; (iv) 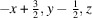; (v) 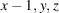.

**Table 2:** Experimental details

Crystal data	
Chemical formula	C_21_H_14_N_2_
*M*_r_	294.34
Crystal system, space group	Orthorhombic, *Pbca*
Temperature (K)	173
*a*, *b*, *c* (Å)	8.30341 (17), 9.5845 (2), 37.0413 (8)
*V* (Å^3^)	2947.90 (11)
*Z*	8
Radiation type	Mo *K*α
μ (mm^−1^)	0.08
Crystal size (mm)	0.11 × 0.10 × 0.01

Data collection	
Diffractometer	Rigaku XtaLAB P200K
Absorption correction	Multi-scan (*CrysAlis PRO*; Rigaku OD, 2024 ▸)
*T*_min_, *T*_max_	0.854, 1.000
No. of measured, independent and observed [*I* > 2σ(*I*)] reflections	59653, 3810, 3005
*R*_int_	0.039
(sin θ/λ)_max_ (Å^−1^)	0.698

Refinement	
*R*[*F*^2^ > 2σ(*F*^2^)], *wR*(*F*^2^), *S*	0.041, 0.103, 1.05
No. of reflections	3810
No. of parameters	212
No. of restraints	1
H-atom treatment	H atoms treated by a mixture of independent and constrained refinement
Δρ_max_, Δρ_min_ (e Å^−3^)	0.23, −0.22

Computer programs: *CrysAlis PRO* (Rigaku OD, 2024 ▸), *SHELXT2018/2* (Sheldrick, 2015*a* ▸), *SHELXL2019/3* (Sheldrick, 2015*b* ▸) and *OLEX2* (Dolomanov *et al.*, 2009 ▸).

## supplementary crystallographic information

### 2-(anthracen-10-yl)-1H-benzo[d]imidazole Crystal data

**Table:**

C_21_H_14_N_2_	*D*_x_ = 1.326 Mg m^−^^3^
*M**_r_* = 294.34	Mo *K*α radiation, λ = 0.71073 Å
Orthorhombic, *P**b**c**a*	Cell parameters from 23539 reflections
*a* = 8.30341 (17) Å	θ = 2.2–28.5°
*b* = 9.5845 (2) Å	µ = 0.08 mm^−^^1^
*c* = 37.0413 (8) Å	*T* = 173 K
*V* = 2947.90 (11) Å^3^	Plate, yellow
*Z* = 8	0.11 × 0.1 × 0.01 mm
*F*(000) = 1232	

### 2-(anthracen-10-yl)-1H-benzo[d]imidazole Data collection

**Table:**

Rigaku XtaLAB P200K diffractometer	3810 independent reflections
Radiation source: Rotating Anode, Rigaku FR-X	3005 reflections with *I* > 2σ(*I*)
Rigaku Osmic Confocal Optical System monochromator	*R*_int_ = 0.039
Detector resolution: 5.8140 pixels mm^-1^	θ_max_ = 29.7°, θ_min_ = 2.2°
shutterless scans	*h* = −10→11
Absorption correction: multi-scan (CrysAlisPro; Rigaku OD, 2024)	*k* = −11→12
*T*_min_ = 0.854, *T*_max_ = 1.000	*l* = −49→50
59653 measured reflections	

### 2-(anthracen-10-yl)-1H-benzo[d]imidazole Refinement

**Table:**

Refinement on *F*^2^	Primary atom site location: dual
Least-squares matrix: full	Hydrogen site location: mixed
*R*[*F*^2^ > 2σ(*F*^2^)] = 0.041	H atoms treated by a mixture of independent and constrained refinement
*wR*(*F*^2^) = 0.103	*w* = 1/[σ^2^(*F*_o_^2^) + (0.0444*P*)^2^ + 0.8465*P*] where *P* = (*F*_o_^2^ + 2*F*_c_^2^)/3
*S* = 1.05	(Δ/σ)_max_ = 0.001
3810 reflections	Δρ_max_ = 0.23 e Å^−^^3^
212 parameters	Δρ_min_ = −0.22 e Å^−^^3^
1 restraint	

### 2-(anthracen-10-yl)-1H-benzo[d]imidazole Special details

**Table:**

Geometry. All esds (except the esd in the dihedral angle between two l.s. planes) are estimated using the full covariance matrix. The cell esds are taken into account individually in the estimation of esds in distances, angles and torsion angles; correlations between esds in cell parameters are only used when they are defined by crystal symmetry. An approximate (isotropic) treatment of cell esds is used for estimating esds involving l.s. planes.
Refinement. Hydrogen atom on N9 was located from the F_map_ and refined isotropically with N—H distance restrained to 0.98 Å

### 2-(anthracen-10-yl)-1H-benzo[d]imidazole Fractional atomic coordinates and isotropic or equivalent isotropic displacement parameters (Å^2^)

**Table:**

	*x*	*y*	*z*	*U*_iso_*/*U*_eq_	
N2	0.84627 (11)	0.24750 (9)	0.66012 (2)	0.0248 (2)	
N9	0.77061 (11)	0.47141 (9)	0.66026 (2)	0.0242 (2)	
H9	0.7166 (17)	0.5562 (14)	0.6545 (4)	0.047 (4)*	
C1	0.75069 (13)	0.34240 (11)	0.64528 (3)	0.0227 (2)	
C3	0.93528 (13)	0.31880 (11)	0.68609 (3)	0.0244 (2)	
C4	1.05794 (14)	0.27224 (12)	0.70899 (3)	0.0309 (3)	
H4	1.092555	0.177772	0.708690	0.037*	
C5	1.12702 (15)	0.36837 (13)	0.73207 (3)	0.0354 (3)	
H5	1.210908	0.339403	0.747813	0.043*	
C6	1.07597 (15)	0.50770 (13)	0.73274 (3)	0.0349 (3)	
H6	1.125182	0.570561	0.749220	0.042*	
C7	0.95616 (14)	0.55626 (12)	0.71013 (3)	0.0303 (3)	
H7	0.922156	0.650883	0.710563	0.036*	
C8	0.88755 (13)	0.45913 (11)	0.68660 (3)	0.0241 (2)	
C10	0.63824 (13)	0.31325 (11)	0.61517 (3)	0.0247 (2)	
C11	0.69459 (14)	0.23639 (11)	0.58494 (3)	0.0281 (2)	
C12	0.85679 (16)	0.18867 (12)	0.58106 (3)	0.0340 (3)	
H12	0.933298	0.209447	0.599360	0.041*	
C13	0.90366 (19)	0.11377 (14)	0.55153 (3)	0.0447 (3)	
H13	1.012312	0.083653	0.549572	0.054*	
C14	0.7934 (2)	0.08031 (15)	0.52384 (4)	0.0514 (4)	
H14	0.826830	0.024926	0.503930	0.062*	
C15	0.6404 (2)	0.12719 (14)	0.52571 (3)	0.0470 (4)	
H15	0.567928	0.106712	0.506574	0.056*	
C16	0.58481 (16)	0.20700 (12)	0.55586 (3)	0.0344 (3)	
C17	0.42855 (16)	0.25784 (14)	0.55726 (3)	0.0381 (3)	
H17	0.357257	0.237922	0.537875	0.046*	
C18	0.37304 (14)	0.33687 (13)	0.58608 (3)	0.0329 (3)	
C19	0.21431 (15)	0.39448 (15)	0.58616 (4)	0.0426 (3)	
H19	0.145176	0.377682	0.566191	0.051*	
C20	0.16066 (15)	0.47227 (16)	0.61405 (4)	0.0447 (3)	
H20	0.056231	0.512637	0.613230	0.054*	
C21	0.26038 (15)	0.49346 (15)	0.64446 (4)	0.0416 (3)	
H21	0.220969	0.546298	0.664275	0.050*	
C22	0.41201 (14)	0.43952 (13)	0.64588 (3)	0.0333 (3)	
H22	0.475211	0.453120	0.666965	0.040*	
C23	0.47781 (13)	0.36291 (11)	0.61628 (3)	0.0275 (2)	

### 2-(anthracen-10-yl)-1H-benzo[d]imidazole Atomic displacement parameters (Å^2^)

**Table:**

	*U*^11^	*U*^22^	*U*^33^	*U*^12^	*U*^13^	*U*^23^
N2	0.0288 (5)	0.0191 (4)	0.0266 (4)	−0.0012 (4)	−0.0007 (4)	0.0005 (3)
N9	0.0247 (5)	0.0190 (4)	0.0290 (5)	0.0010 (4)	−0.0006 (4)	−0.0015 (4)
C1	0.0237 (5)	0.0191 (5)	0.0254 (5)	−0.0020 (4)	0.0031 (4)	−0.0004 (4)
C3	0.0275 (5)	0.0218 (5)	0.0238 (5)	−0.0018 (4)	0.0017 (4)	0.0000 (4)
C4	0.0351 (6)	0.0277 (6)	0.0300 (6)	0.0025 (5)	−0.0030 (5)	0.0034 (4)
C5	0.0361 (6)	0.0402 (7)	0.0301 (6)	0.0002 (5)	−0.0074 (5)	0.0026 (5)
C6	0.0361 (6)	0.0377 (7)	0.0309 (6)	−0.0049 (5)	−0.0033 (5)	−0.0081 (5)
C7	0.0324 (6)	0.0263 (5)	0.0323 (6)	−0.0019 (5)	0.0015 (5)	−0.0061 (4)
C8	0.0247 (5)	0.0232 (5)	0.0244 (5)	−0.0006 (4)	0.0026 (4)	−0.0002 (4)
C10	0.0284 (5)	0.0198 (5)	0.0259 (5)	−0.0034 (4)	−0.0007 (4)	0.0018 (4)
C11	0.0372 (6)	0.0212 (5)	0.0258 (5)	−0.0031 (5)	0.0000 (5)	0.0017 (4)
C12	0.0426 (7)	0.0297 (6)	0.0297 (6)	0.0054 (5)	0.0032 (5)	0.0013 (5)
C13	0.0597 (9)	0.0393 (7)	0.0351 (7)	0.0149 (7)	0.0094 (6)	0.0012 (5)
C14	0.0829 (12)	0.0400 (8)	0.0313 (7)	0.0077 (8)	0.0089 (7)	−0.0089 (6)
C15	0.0705 (10)	0.0422 (8)	0.0282 (6)	−0.0088 (7)	−0.0035 (6)	−0.0067 (5)
C16	0.0476 (7)	0.0286 (6)	0.0269 (5)	−0.0081 (5)	−0.0021 (5)	−0.0002 (4)
C17	0.0431 (7)	0.0407 (7)	0.0304 (6)	−0.0126 (6)	−0.0104 (5)	0.0020 (5)
C18	0.0316 (6)	0.0342 (6)	0.0330 (6)	−0.0087 (5)	−0.0044 (5)	0.0076 (5)
C19	0.0295 (6)	0.0566 (8)	0.0418 (7)	−0.0082 (6)	−0.0080 (5)	0.0138 (6)
C20	0.0242 (6)	0.0577 (9)	0.0523 (8)	0.0019 (6)	0.0020 (6)	0.0157 (7)
C21	0.0299 (6)	0.0487 (8)	0.0462 (7)	0.0008 (6)	0.0076 (6)	0.0009 (6)
C22	0.0264 (6)	0.0388 (7)	0.0347 (6)	−0.0036 (5)	0.0021 (5)	0.0002 (5)
C23	0.0283 (5)	0.0248 (5)	0.0294 (5)	−0.0054 (4)	−0.0014 (4)	0.0042 (4)

### 2-(anthracen-10-yl)-1H-benzo[d]imidazole Geometric parameters (Å, º)

**Table:**

N2—C1	1.3263 (14)	C12—C13	1.3653 (17)
N2—C3	1.3924 (14)	C13—H13	0.9500
N9—H9	0.952 (13)	C13—C14	1.412 (2)
N9—C1	1.3653 (13)	C14—H14	0.9500
N9—C8	1.3816 (14)	C14—C15	1.350 (2)
C1—C10	1.4812 (14)	C15—H15	0.9500
C3—C4	1.3986 (16)	C15—C16	1.4301 (17)
C3—C8	1.4023 (15)	C16—C17	1.3869 (19)
C4—H4	0.9500	C17—H17	0.9500
C4—C5	1.3815 (17)	C17—C18	1.3876 (18)
C5—H5	0.9500	C18—C19	1.4290 (18)
C5—C6	1.4013 (18)	C18—C23	1.4389 (15)
C6—H6	0.9500	C19—H19	0.9500
C6—C7	1.3812 (17)	C19—C20	1.350 (2)
C7—H7	0.9500	C20—H20	0.9500
C7—C8	1.3968 (15)	C20—C21	1.4127 (19)
C10—C11	1.4196 (15)	C21—H21	0.9500
C10—C23	1.4152 (16)	C21—C22	1.3620 (18)
C11—C12	1.4296 (17)	C22—H22	0.9500
C11—C16	1.4390 (16)	C22—C23	1.4283 (16)
C12—H12	0.9500		
			
N2···C12	2.9836 (13)	C1···H12	2.61
N9···N2^i^	2.8185 (12)	C1···H22	2.65
N9···C22	3.0403 (15)	C6···H21^ii^	2.83
N2···H12	2.39	C7···H21^ii^	2.78
H9···N2^i^	1.918 (13)	C22···H9	2.784 (14)
N9···H22	2.47	H9···H22	2.28
H9···C1^i^	2.778 (13)		
			
C1—N2—C3	105.51 (9)	C12—C13—H13	119.4
C1—N9—H9	128.7 (9)	C12—C13—C14	121.12 (14)
C1—N9—C8	107.15 (9)	C14—C13—H13	119.4
C8—N9—H9	124.2 (9)	C13—C14—H14	120.1
N2—C1—N9	112.34 (9)	C15—C14—C13	119.85 (12)
N2—C1—C10	124.05 (9)	C15—C14—H14	120.1
N9—C1—C10	123.59 (9)	C14—C15—H15	119.3
N2—C3—C4	130.47 (10)	C14—C15—C16	121.46 (13)
N2—C3—C8	109.27 (9)	C16—C15—H15	119.3
C4—C3—C8	120.24 (10)	C15—C16—C11	119.01 (12)
C3—C4—H4	121.2	C17—C16—C11	119.72 (11)
C5—C4—C3	117.70 (11)	C17—C16—C15	121.26 (12)
C5—C4—H4	121.2	C16—C17—H17	119.0
C4—C5—H5	119.3	C16—C17—C18	122.10 (11)
C4—C5—C6	121.40 (11)	C18—C17—H17	119.0
C6—C5—H5	119.3	C17—C18—C19	121.26 (11)
C5—C6—H6	119.1	C17—C18—C23	119.46 (11)
C7—C6—C5	121.88 (11)	C19—C18—C23	119.27 (11)
C7—C6—H6	119.1	C18—C19—H19	119.3
C6—C7—H7	121.7	C20—C19—C18	121.30 (12)
C6—C7—C8	116.59 (11)	C20—C19—H19	119.3
C8—C7—H7	121.7	C19—C20—H20	120.1
N9—C8—C3	105.72 (9)	C19—C20—C21	119.75 (12)
N9—C8—C7	132.11 (10)	C21—C20—H20	120.1
C7—C8—C3	122.17 (10)	C20—C21—H21	119.4
C11—C10—C1	118.95 (10)	C22—C21—C20	121.20 (13)
C23—C10—C1	120.53 (9)	C22—C21—H21	119.4
C23—C10—C11	120.51 (10)	C21—C22—H22	119.4
C10—C11—C12	123.76 (10)	C21—C22—C23	121.27 (12)
C10—C11—C16	118.89 (11)	C23—C22—H22	119.4
C12—C11—C16	117.33 (10)	C10—C23—C18	119.22 (10)
C11—C12—H12	119.4	C10—C23—C22	123.73 (10)
C13—C12—C11	121.15 (12)	C22—C23—C18	117.05 (11)
C13—C12—H12	119.4		
			
N2—C1—C10—C11	−46.69 (15)	C10—C11—C16—C17	2.15 (16)
N2—C1—C10—C23	134.18 (11)	C11—C10—C23—C18	−1.16 (16)
N2—C3—C4—C5	179.14 (11)	C11—C10—C23—C22	179.29 (10)
N2—C3—C8—N9	−0.86 (11)	C11—C12—C13—C14	−0.3 (2)
N2—C3—C8—C7	179.87 (10)	C11—C16—C17—C18	−0.14 (18)
N9—C1—C10—C11	131.47 (11)	C12—C11—C16—C15	2.81 (16)
N9—C1—C10—C23	−47.65 (15)	C12—C11—C16—C17	−176.28 (11)
C1—N2—C3—C4	−177.46 (11)	C12—C13—C14—C15	2.5 (2)
C1—N2—C3—C8	0.92 (11)	C13—C14—C15—C16	−2.0 (2)
C1—N9—C8—C3	0.46 (11)	C14—C15—C16—C11	−0.7 (2)
C1—N9—C8—C7	179.63 (12)	C14—C15—C16—C17	178.41 (13)
C1—C10—C11—C12	−2.26 (16)	C15—C16—C17—C18	−179.21 (12)
C1—C10—C11—C16	179.42 (9)	C16—C11—C12—C13	−2.37 (17)
C1—C10—C23—C18	177.95 (10)	C16—C17—C18—C19	176.68 (12)
C1—C10—C23—C22	−1.60 (16)	C16—C17—C18—C23	−2.53 (18)
C3—N2—C1—N9	−0.64 (12)	C17—C18—C19—C20	−179.47 (13)
C3—N2—C1—C10	177.71 (9)	C17—C18—C23—C10	3.16 (16)
C3—C4—C5—C6	0.28 (18)	C17—C18—C23—C22	−177.26 (11)
C4—C3—C8—N9	177.72 (10)	C18—C19—C20—C21	−2.4 (2)
C4—C3—C8—C7	−1.55 (16)	C19—C18—C23—C10	−176.07 (11)
C4—C5—C6—C7	−0.92 (19)	C19—C18—C23—C22	3.51 (16)
C5—C6—C7—C8	0.31 (18)	C19—C20—C21—C22	1.6 (2)
C6—C7—C8—N9	−178.15 (11)	C20—C21—C22—C23	1.8 (2)
C6—C7—C8—C3	0.91 (16)	C21—C22—C23—C10	175.24 (11)
C8—N9—C1—N2	0.12 (12)	C21—C22—C23—C18	−4.32 (17)
C8—N9—C1—C10	−178.24 (9)	C23—C10—C11—C12	176.86 (10)
C8—C3—C4—C5	0.91 (16)	C23—C10—C11—C16	−1.46 (16)
C10—C11—C12—C13	179.28 (11)	C23—C18—C19—C20	−0.26 (19)
C10—C11—C16—C15	−178.76 (11)		

Symmetry codes: (i) −*x*+3/2, *y*+1/2, *z*; (ii) *x*+1, *y*, *z*.

### 2-(anthracen-10-yl)-1H-benzo[d]imidazole Hydrogen-bond geometry (Å, º)

*Cg*1, *Cg*2, *Cg*3 and *Cg*4 are the centroids of the (N2/N9/C1/C3–C8), (C10/C11/C16–C21), (C10/C11/C16–C18,C23) and (C3–C8) rings, respectively.

**Table:**

*D*—H···*A*	*D*—H	H···*A*	*D*···*A*	*D*—H···*A*
N9—H9···N2^i^	0.95 (1)	1.92 (1)	2.8186 (13)	157 (1)
C5—H5···*Cg*1^iii^	0.95	2.96	3.8219 (12)	151
C13—H13···*Cg*2^iv^	0.95	2.97	3.4156 (14)	110
C20—H20···*Cg*3^i^	0.95	2.90	3.5625 (16)	128
C21—H21···*Cg*4^v^	0.95	2.75	3.2887 (15)	116

Symmetry codes: (i) −*x*+3/2, *y*+1/2, *z*; (iii) *x*+1/2, *y*, −*z*+3/2; (iv) −*x*+3/2, *y*−1/2, *z*; (v) *x*−1, *y*, *z*.
